# Association between Levels of Physical Activity, Sarcopenia, Type 2 Diabetes and the Quality of Life of Elderly People in Community Dwellings in Lebanon

**DOI:** 10.3390/geriatrics6010028

**Published:** 2021-03-18

**Authors:** Dana Saadeddine, Leila Itani, Dima Kreidieh, Dana El Masri, Hana Tannir, Marwan El Ghoch

**Affiliations:** Department of Nutrition and Dietetics, Faculty of Health Sciences, Beirut Arab University, P.O. Box 11-5020 Riad El Solh, Beirut 11072809, Lebanon; dana_saadeddine_94@hotmail.com (D.S.); l.itani@bau.edu.lb (L.I.); d.kraydeyeh@bau.edu.lb (D.K.); dana.masri@bau.edu.lb (D.E.M.); hana.tannir@bau.edu.lb (H.T.)

**Keywords:** physical activity, body composition, sarcopenia, sedentary lifestyle, type 2 diabetes

## Abstract

There is a lack of data from developing countries on the link between physical activity (PA) on health outcomes. This study examines the association between the level of PA and sarcopenia, cardiovascular risk factors (i.e., dyslipidemia, type 2 diabetes (T2D), and cardiovascular diseases), and the health-related quality of life (HRQoL) among elderly people, in community dwellings in Lebanon. In this cross-sectional, observational study, body composition, levels of PA, and the HRQoL of 243 elderly people living in community dwellings, are obtained. The participants are then categorized based on a PA cut-off point of 600 metabolic equivalent task minutes per week (MET-min/week). In our sample, the prevalence of physical inactivity, defined as performing less than 600 MET-min/week, is 51.44% (125/243 participants).They displayed a higher prevalence of sarcopenia (36.0% vs. 18.6%), T2D (39.6% vs. 21.1%), as well as a lower physical (65.67 ± 20.72 vs. 75.08 ± 17.29) and mental (67.58 ± 21.51 vs. 76.95 ± 17.16) HRQoL. On the other hand, regression analysis shows that an increased rate of PA to ≥600 MET-min/week is associated with a lower risk of T2D (OR = 0.43, 95% CI: 0.22–0.84, *p* = 0.013) and sarcopenia (OR= 0.40, 95% CI: 0.22–0.73, *p* = 0.003) by 60%, and higher scores of the physical (β = −7.65; −11.87, −3.43, *p* = 0.0004) and mental (β = −8.47; −13.08, −3.85, *p* = 0.0004) HRQoL by nearly eight points. Our results show a high prevalence of physical inactivity in Lebanese adults over the age of 60; however, an adequate level of PA among this population seemed to be associated with a lower risk of sarcopenia and T2D, as well as a better HRQoL. However, future longitudinal studies are still needed to clarify if intervention based on increasing levels of PA can determine improvement in these clinical outcomes. If this is shown to be the case, it emphasizes the importance of implementing strategies to increase physical activity within this population.

## 1. Introduction

Physical activity (PA) is defined as any movement of the body through the skeletal muscle that results in an increase in energy expenditure [1]. The World Health Organization (WHO) updated global guidelines on PA and sedentary behavior for children, adolescents, adults, and older adults, which were released in 2020 [2]. The development of these guidelines provides a set of evidence-based recommendations that governments can adopt as part of their national policy frameworks to support comprehensive approaches to increasing levels of PA among the population; a global action plan on physical activity between 2018–2030 aims to reduce physical inactivity by 15% by 2030 [2].

A large quantity of data, sourced primarily from Western societies, relates PA to health outcomes across an entire lifespan [3,4,5,6]. In particular, the benefits of PA have become evident among older adults in several clinical outcomes, i.e., the reduction in mortality of all-cause and cardiovascular disease, a lower incidence of non-communicable diseases (NCDs), an enhancement of mental health and functional ability, and the prevention of falls and fall-related injuries, prevalent among this population [2,3,7]. For this reason and according to the WHO global recommendation of PA for health in adults to do to at least 150 min of moderate-intensity, aerobic physical activity or at least 75 min of vigorous intensity, aerobic PA or an equivalent combination of moderate- and vigorous-intensity activity per week equivalent to ≥600 MET (metabolic equivalent task)-min/week [2,8].

Despite this fact, few studies have been conducted on older adults living in Arab-speaking countries, Reference [9] and those few available studies, conducted on this specific population, are composed of small samples [10], with inconsistent use of validated tools to define and assess levels of PA [10,11]. In addition, there is a lack of standardized guidelines for physical inactivity quantification and interpretation for the population from the Middle East and North Africa (MENA) region [12], especially middle-income countries [13], which appear to be much needed and form the basis of our study.

Accordingly, we aimed to assess the association between these cut-off points relating to the amount of PA (≥600 MET-min/week) with three important health-related outcomes, that have recently been shown to be independent predictors of mortality among older adults in community dwellings [14,15,16], namely, sarcopenia [14], cardiovascular risk factors [15], and HRQoL [16]. The hypothesis that we formulate is the existence of a significant association between suitable levels of PA and a lower risk of sarcopenia and cardiovascular risk factors, as well as better HRQoL among elderly people in community dwellings in Lebanon.

## 2. Materials and Methods

The study was conducted in the Department of Nutrition and Dietetics at Beirut Arab University (BAU) in Lebanon, during the period March 2018–February 2020. A total of 243 participants, both male and female, were consecutively recruited from the general population through a simple, randomized, community e-mail-based survey, sent to members of BAU and other mailing lists, and focusing on elderly people of ≥60 years old, living independently in community dwellings. This group can suffer from an extensive range of health care problems, from age-related issues (not disease specific) to suffering from multiple pathologies. The inclusion criteria were (i) age ≥ 60 years old and (ii) having the ability to read and write, in order to follow instructions regarding how to fill out the questionnaires. The exclusion criteria were (i) the inability to move without crutches, a walker or other assistive devices, (ii) the presence of artificial limbs or limb prosthesis, (iii) the presence of severe cognitive impairment (i.e., dementia, Alzheimer’s disease), active cancer, congestive heart failure, a chronic obstructive pulmonary disorder, chronic renal failure, cirrhosis or liver failure.

The study was approved by the Institutional Review Board of BAU, (No. 2019H-0063-HS-M-0318), and all participants gave informed, written consent for the use of their anonymized personal data. A questionnaire was administered to the participants, to elicit information regarding medical history and lifestyle, as well as demographic and social conditions.

Bodyweight was measured by a trained dietician involved in the study, using an electronic weighing scale (SECA 2730-ASTRA, Germany). Height was measured using a stadiometer. BMI was then calculated according to the standard formula; bodyweight measured in kilograms, divided by the square of the height in meters.

On the same occasion, a multi-frequency, segmental, body composition analyzer (MC-780MA, Tanita Corp., Tokyo, Japan) was used to measure body composition, based on three frequencies and providing highly accurate, whole-body and segmental measurements [17,18]. Participant age, sex, and height information were entered into the device [17,18]. Then, the participant was instructed to stand in a stable, barefoot position. Separate readings for different body segment compositions were obtained. These readings were based on an algorithm incorporating impedance, age, and height [17,18]. All recommendations for a correct bioimpedance (BIA) measurement were followed, such as taking measurements more than 3 h after waking, urinating before the measurement, not consuming food or drink for at least 8 h previously, not exercising during the 12 h prior to measurement and not consuming alcohol or energy drinks in the previous 12 h. Participants should not have had any metal plates or rods inserted, neither should they have had a pacemaker fitted. According to the literature, this particular BIA model (MC-780MA, Tanita Corp., Tokyo, Japan), when compared with DXA used in other published studies [17,18], showed excellent reproducibility in assessing overweight and obese individuals, regardless of their level of physical activity [17,18]. Total fat, lean mass percentages, and the appendicular lean mass (ALM) were calculated, using standard formulas. Sarcopenia was defined based on the definition of Oh and colleagues ((ALM/weight) × 100%), which was less than 23.40 and 29.60 in females and males, respectively [19].

The short validated Arabic version of the SF-36 questionnaire [20] was used to assess the participants’ HRQoL, which comprised 36 questions and an eight-scale profile of functional health and well-being scores: Physical functioning, role limitations (due to physical problems), bodily pain, general health, vitality, social functioning, role limitations (due to emotional problems), mental health. The guidelines for calculation of the SF-36 were used to calculate the sub scores [21]. The score ranged between 0 and 100, with a higher score indicating a higher level of function and/or better health, and a lower score indicating a lower level of function and/or poor health. Two summary measures were calculated, namely, a physical health score (physical functioning, role limitation (due to physical health), bodily pain, and general health) and a mental health score (vitality, social functioning, role limitation (due to emotional problems), mental health) by averaging the component scores from the corresponding subscales [21].

Subjective PA was assessed using the official, self-administrated Arabic short-version format of the IPAQ that is available at www.ipaq.ki.se and used elsewhere in other Arabic populations [22]. The IPAQ is an instrument, designed primarily for surveillance of PA among the population [23]. It covers three domains of PA: Walking, moderate-, and vigorous-intensity activities. The questionnaire also includes questions relating to time spent sitting, as an indicator of sedentary behavior [23]. In each of the four domains, the number of days per week and the time spent per day participating in both moderate and vigorous activity or sedentary behavior over the last seven days are recorded. IPAQ is considered to have reasonable measurement properties for monitoring levels of PA among the population in diverse settings [23,24]. Responses were converted to MET-min/week, Reference [23] according to the IPAQ scoring protocol: Total minutes over the last seven days spent on vigorous activity, moderate-intensity activity, and walking were multiplied by 8.0, 4.0, and 3.3, respectively, to create MET scores for each activity level. The MET scores across the three sub-components were summed to indicate overall physical activity [23]. The total physical activity of ≥600 MET-min/week denotes adequate physical activity or being physically active, and an inadequate level of physical activity or physical inactivity was represented by <600 MET-min/week [8].

Cardiovascular risk factors in this study indicate the presence of any diseases, such as type 2 diabetes, cardiovascular diseases (hypertension, coronary heart disease, stroke, transient ischemic attack, and peripheral arterial disease), and dyslipidemia (a decreased concentration of high-density lipoprotein cholesterol and an increased concentration of high-density lipoprotein cholesterol and triglycerides), based on self-reported diagnosis, either simultaneously or separately.

### Statistical Analysis

The descriptive statistics are presented as mean and standard deviations for continuous variables and frequencies, and proportions for categorical variables. The student t-test was used for mean comparison, and the Chi-squared test for independence was used to test the association between categorical variables. Univariate and multivariate logistic models were used to determine the odds ratio of having sarcopenia and cardiovascular risk factors, as dependent variables, with higher physical activity levels (MET-min/week ≥ 600) as an independent variable. Univariate and multivariate Linear regression models were used to determine the effect size (β coefficient) of Lower physical activity (MET-min/week <600), as an independent variable, on physical and mental quality of life (used as the linear scores of the SF-36 subscales), as dependent variables, respectively. In both types of regression, models were adjusted for potential confounders (i.e., age, sex, having obesity or sarcopenia or not), considering a *p* value < 0.25 in the univariate model [25]. The statistical significance for all tests was set at *p* < 0.05. All statistical analysis was done using SPSS ver. 26 (Armonk, NY: IBM Corp, 2019).

## 3. Results

The characteristics of the study population are shown in Table 1. The mean age, sex, social status and income did not vary across the two PA categories (≥600 vs. <600 MET-min/week). The participants with lower PA levels constituted 125 of the total 243 participants, and account for 51.44% of the entire sample. These individuals were more likely to have a higher BMI (31.77 ± 5.58 vs. 29.81 ± 4.42Kg/m^2^; *p* = 0.003), a lower standard of education (75.2% vs. 50.8%; *p* < 0.0001), poorer physical health (65.67 ± 20.72 vs. 75.08 ± 17.29; *p* < 0.0001), poorer mental health (67.58 ± 21.51 vs. 76.95 ± 17.16; *p* = 0.0001) and were more likely to have sarcopenia (36.0% vs. 18.6%; *p* = 0.002), and type 2 diabetes (39.6% vs. 21.1%; *p* = 0.006).

Logistic regression analysis showed that increasing the rate of PA to ≥ 600 Met-min/week is associated with lower risk of T2D (OR = 0.43, 95%CI: 0.22–0.84, *p* = 0.013) by 60% (Table 2) and sarcopenia by 60% (OR = 0.40, 95%CI: 0.22–0.73 *p* = 0.003) (Table 3). Furthermore, linear regression analysis showed that both the SF-36 physical health score (β = −7.65; −11.87, −3.43, *p* = 0.0004) and the mental health score (β = −8.47; −13.08, −3.85, *p* = 0.0004) are lower by nearly eight points if the individual was not physically active (MET-min/week < 600) (Table 4 and Table 5).

## 4. Discussion

**Figure 1:**
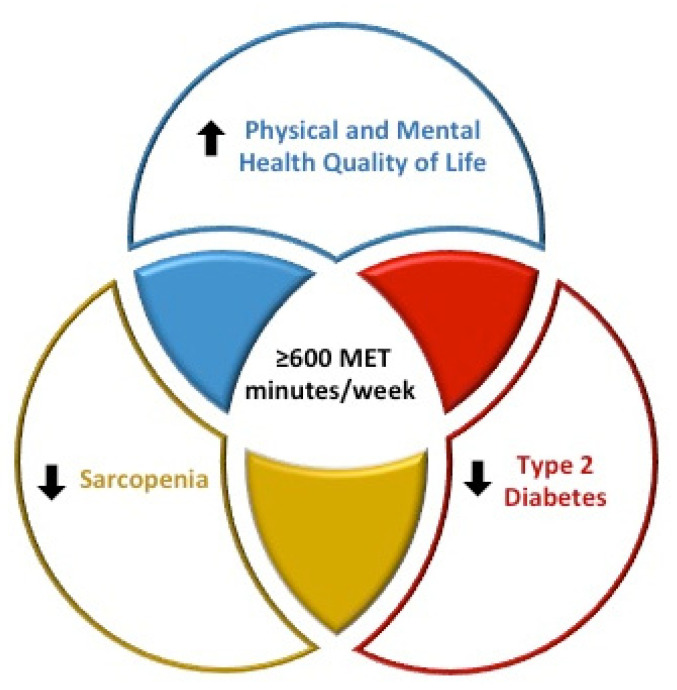
Diagram physical activity, sarcopenia, type 2 diabetes, and health-related quality of life.

Little is known about PA and its impact on key health outcomes among older Lebanese adults. For this reason, our study aimed to provide data relating to the association between PA levels and sarcopenia, a reduction of cardiovascular risk factors, and an improvement in HRQoL. In turn, four major findings were revealed (Figure 1).

### 4.1. Findings and Concordance with Previous Studies

Firstly, the main finding of our study is that older male and female adults, who participate in an adequate level of PA (i.e., ≥600 MET-min/week as a cut-off, which relates to at least 150 min/week of moderate-intensity aerobic physical activity or at least 75 min/week of vigorous-intensity aerobic physical activity) seem to be less likely to be affected by sarcopenia. Our finding is in line with robust data deriving from a recent systematic review, that showed a significant inverse association between levels of PA and sarcopenia [26]. However, none of the studies included in this systematic review, was conducted in Arab speaking countries [26]. Moreover, to the best of our knowledge, only one recent study assessed the association between PA patterns and sarcopenia among Arabs in Saudi Arabia; however, the sample included in this study comprised young and only male adults [27]. The authors in this study concluded that future studies should conduct an investigation among the older population [27].

Secondly, the individuals in our study who reported levels of PA of ≥600 MET-min/week to seem to be better protected from cardiovascular risk factors, in particular, type 2 diabetes. Despite this fact, our finding may not appear new, since (on a global scale) it has been well established that regular PA improves the glycemic blood profile, and can also delay or completely prevent type 2 diabetes [28], however, we consider it useful to highlight this finding specifically in our population. Thirdly, higher levels of PA (≥600 MET-min/week) were associated with a better HRQoL, which emphasizes the importance of increasing levels of PA among this population. This finding is in line with previous studies conducted among other populations (not Arabic or Lebanese) [29].

Finally, nearly half of our sample (51.44%) was categorized as physically inactive, defined as partaking in <600 MET-min/week, and that widely exceed the prevalence of physical inactivity in older Europeans (≥55 years old) has been reported to range from 5% in Sweden to 29% in Portugal [30]. Moreover, our findings are not in accordance with the limited studies that included samples similar to ours (i.e., age > 60) [10,11]. In fact, the reported prevalence of PA in these studies exceeded 60% [10,11]. The first study was conducted in Jeddah (Saudi Arabia) on a very limited sample, composed of 55 adults (>60 years old), indicating a prevalence of individuals of 69%, characterized by physical inactivity [10]. The second study has been conducted on 340 adults over the age of 60 and living in Urban Suez (Egypt); the study reported a prevalence of physical inactivity of 63.8% [11]. However, in both studies, there was uncertainty as to which instrument was used to assess levels of PA, and the definition and cut-offs of physical inactivity were also unclear [10,11]. We speculate that the shortcomings in these two studies [10,11] may have resulted in the discrepancies between our findings and those of the limited studies.

### 4.2. Potential Clinical Implications

From these three findings, certain clinical implications can be deduced. Foremost, the awareness among health professionals, as well as individuals in the general population, of the high prevalence of physical inactivity among the older Lebanese adult population. It is also vital that health professionals openly discuss with older Lebanese adults in the different settings the association between adequate levels of PA (≥600 MET-min/week), and lower risk of sarcopenia and T2D, and higher scores of physical- and mental- HRQoL (Figure 1).

### 4.3. Study Strengths

Our study has certain strengths. Principally, to the best of our knowledge, it is one of the few studies which assesses the association of levels of PA and three important clinical outcomes, shown to be predictors of mortality among older adults in community dwellings [14,15,16]. Secondly, the instrument used for the assessment of HRQoL was a validated questionnaire, suitable for the population under study [20]. Finally, we used the Oh definition for sarcopenia [19], which has been revealed to be of clinical relevance for the Lebanese population [31,32,33].

### 4.4. Study Limitations

However, our study did have certain limitations. Firstly, the information relative to PA and cardiovascular risk factors were self-reported and did not rely on objective assessments (i.e., accelerometers, biochemical blood tests, etc.), and this may determine certain discrepancies between the measured and reported data [34]. Moreover, the use of IPAQ may not be the most suitable questionnaire for the assessment of subjective PA in older adults, since other PA questionnaires, specifically designed for the elderly, are available (i.e., The Physical Activity Scale for the Elderly (PASE)) [35]. Secondly, we assessed body composition using the impedance analyzer; despite its validation vs. Dual-Energy X-Ray Absorptiometry (DXA) scans, it is still not accepted as a gold-standard technique. However, multi-frequency BIA has been found to be a very accurate measurement; it correlates with DXA in healthy adults [36,37], and has been widely validated in overweight and obese individuals in several clinical settings [38,39]. Thirdly, in relation to the cross-sectional design, no causal associations could be inferred between levels of PA and clinical outcomes, and the small sample of our study should be considered with regard to other limitations.

## 5. Conclusions

In conclusion, a high prevalence of physical inactivity was noticed in Lebanese adults over the age of 60; however, suitable levels of PA seem to be associated with a lower prevalence of sarcopenia, a reduced T2D, and a better quality of life. However, future longitudinal studies are still needed to clarify if interventions based on increasing levels of PA can determine improvement in these clinical outcomes (i.e., sarcopenia, T2D, and HRQoL). If this is shown to be the case, it emphasizes the importance of implementing strategies to increase PA among this population, such as reaching suitable levels of PA, i.e., ≥ 600 MET-minute/week, which corresponds to at least 150 min of moderate-intensity PA or 75 min of vigorous-intensity PA weekly.

## Figures and Tables

**Table 1:** Socio-demographic, anthropometric and cardiovascular risk factors of the study population (*n* = 243).

		MET-min/Week		
	Total *n* = 243	<600 *n* = 125	≥600 *n* = 118	Significance
Age (years)	67.87 (6.64)	68.27 (6.63)	67.44 (6.65)	*p* = 0.328
BMI (Kg/m^2^)	30.79 (5.14)	31.77 (5.58)	29.81 (4.42)	*p* = 0.003
ALM/Weight × 100%	27.77 (3.52)	27.13 (3.38)	28.44 (3.55)	*p* = 0.004
Level of PA (MET-min/week)	1294.22 (2266.70)	230.33 (195.51)	2421.22 (2845.42)	*p* < 0.0001
Sex				X^2^ = 1.832; *p* = 0.176
Male	123 (50.6)	58 (46.4)	65 (55.1)	
Female	120 (49.4)	67 (53.6)	53 (44.9)	
Marital status				X^2^ = 2.610; *p* = 0.106
Unmarried	78 (32.1)	46 (36.8)	32 (27.1)	
Married	165 (67.9)	79 (63.2)	86 (72.9)	
Level of education				X^2^ = 15.591; *p* < 0.0001
Primary	154 (63.4)	94 (75.2)	60 (50.8)	
High school	45 (18.5)	15 (12.0)	30 (25.4)	
University	44 (18.1)	16 (12.8)	28 (23.7)	
Monthly Salary				X^2^ = 1.989; *p* = 0.354
<1 million	167 (68.7)	91 (72.8)	76 (64.4)	
>1 million	76 (31.3)	34 (27.2)	42 (35.6)	
Employment				X^2^ = 0.746; *p* = 0.388
No	193 (79.4)	102 (81.6)	91 (77.1)	
Yes	50 (20.6)	23 (18.4)	27 (22.9)	
Smoking				X^2^ = 1.857; *p* = 0.173
No	150 (61.7)	72 (57.6)	78 (66.1)	
Yes	93 (38.3)	53 (42.4)	40 (33.9)	
Obesity (≥30 Kg/m^2^)				X^2^ = 3.965; *p* = 0.046
No	122 (50.2)	55 (44.0)	67 (56.8)	
Yes	121 (49.8)	70 (56.0)	51 (43.2)	
Sarcopenia				X^2^ = 9.156; *p* = 0.002
No	176 (72.4)	80 (64.0)	96 (81.4)	
Yes	67 (27.6)	45 (36.0)	22 (18.6)	
Dyslipidemia				X^2^ = 2.619; *p* = 0.106
No	127 (68.3)	57 (62.6)	70 (73.7)	
Yes	59 (31.7)	34 (37.4)	25 (26.3)	
Type 2 Diabetes				X^2^ = 7.566; *p* = 0.006
No	130 (69.0)	55 (60.4)	75 (78.9)	
Yes	56 (30.1)	36 (39.6)	20 (21.1)	
Cardiovascular disease				X^2^ = 2.649; *p* = 0.104
No	89 (47.8)	38 (41.8)	51 (53.7)	
Yes	97 (52.2)	53 (58.2)	44 (46.3)	
SF-36				
Physical health	70.24 (19.67)	65.67 (20.72)	75.08 (17.29)	*p* < 0.0001
Mental health	72.13 (20.04)	67.58 (21.51)	76.95 (17.16)	*p* < 0.0001

Values are Means (SD) for continuous variables and n (%) for categorical variables; *p* = level of statistical significance; X^2^ = Chi-Square; BMI = Body mass index; PA = physical activity; ALM = appendicular lean mass.

**Table 2:** The odds ratio of type 2 diabetes among study participants with physical activity level ≥ 600 MET-min/week.

	OR (95%CI)	
	Univariate Model *	Multivariate Model *
Age (years)	1.04 (0.99; 1.09)	1.05 (1.00; 1.11)
Sex		
Male	1	1
Female	1.23 (0.66; 2.31)	1.06 (0.55; 2.05)
Body mass index (Kg/m^2^)		
Without obesity	1	1
With obesity	2.18 (1.15; 4.12)	2.26 (1.15; 4.44)
MET-min/week		
<600	1	1
≥600	0.41 (0.21; 0.78)	0.43 (0.22; 0.84)

* The Univariate model presents the Odds of each independent variable entered in the model separately; the Multivariate models are adjusted for age, sex, and BMI categories.

**Table 3:** The odds ratio of sarcopenia among study participants with physical activity level ≥ 600 MET-min/week.

	OR (95%CI)	
	Univariate Model *	Multivariate Model *
Age (years)	1.00 (0.96; 1.05)	1.00 (0.96; 1.04)
Sex		
Male	1	1
Female	0.91 (0.52; 1.61)	0.84 (0.47; 1.50)
MET-min/week		
<600	1	1
≥600	0.41 (0.23; 0.74)	0.40 (0.22; 0.73)

* The Univariate model presents the Odds of each independent variable entered in the model separately; the Multivariate models are adjusted for age, sex.

**Table 4:** Linear regression coefficients for the association of lower physical activity level with physical health quality of life.

	Univariate Model *		Multivariate Model *	
	β Coefficient			
	Unstandardized β (95%CI)	Standardized	Unstandardized β (95%CI)	Standardized
Age (years)	−0.25 (−0.63; 0.12)	−0.09	−0.14 (−0.46; 0.17)	−0.05
Male	21.13 (16.93; 25.33)	0.54	20.43 (16.23; 24.64)	0.52
With obesity	−3.32 (−8.28; 1.64)	−0.09	0.41 (−4.23; 5.05)	0.01
Sarcopenia	−0.60 (−6.18; 4.97)	−0.01	0.43 (−4.73; 5.59)	0.01
MET-min/week < 600	−9.42 (−14.25; −4.58)	−0.24	−7.65 (−11.87; −3.43)	−0.20

* The Univariate model presents the β coefficient of each independent variable entered in the model separately; the Multivariate models are adjusted for age, sex, having obesity, and the presence of sarcopenia.

**Table 5:** Linear regression coefficients for the association of lower physical activity level with mental health quality of life.

	Univariate Model *		Multivariate Model *	
	β Coefficient			
	Unstandardized β (95%CI)	Standardized	Unstandardized β (95%CI)	Standardized
Age (years)	−0.11 (−0.49; 0.28)	−0.04	−0.10 (−0.33; 0.35)	0.003
Male	17.12 (12.54; 21.71)	0.428	16.78 (12.19; 21.38)	0.420
With obesity	−0.54 (−0.56; 4.54)	−0.013	2.68 (-2.39; 7.75)	0.067
Sarcopenia	0.95 (−4.73; 6.63)	0.021	1.16 (−4.48; 6.80)	0.026
MET-min/week < 600	−9.37 (−14.30; −4.43)	−0.23	−8.47 (−13.08; −3.85)	−0.212

* The Univariate model presents the β coefficient of each independent variable entered in the model separately; the Multivariate models are adjusted for age, sex, having obesity, and the presence of sarcopenia.

## Data Availability

Data are available upon request.
